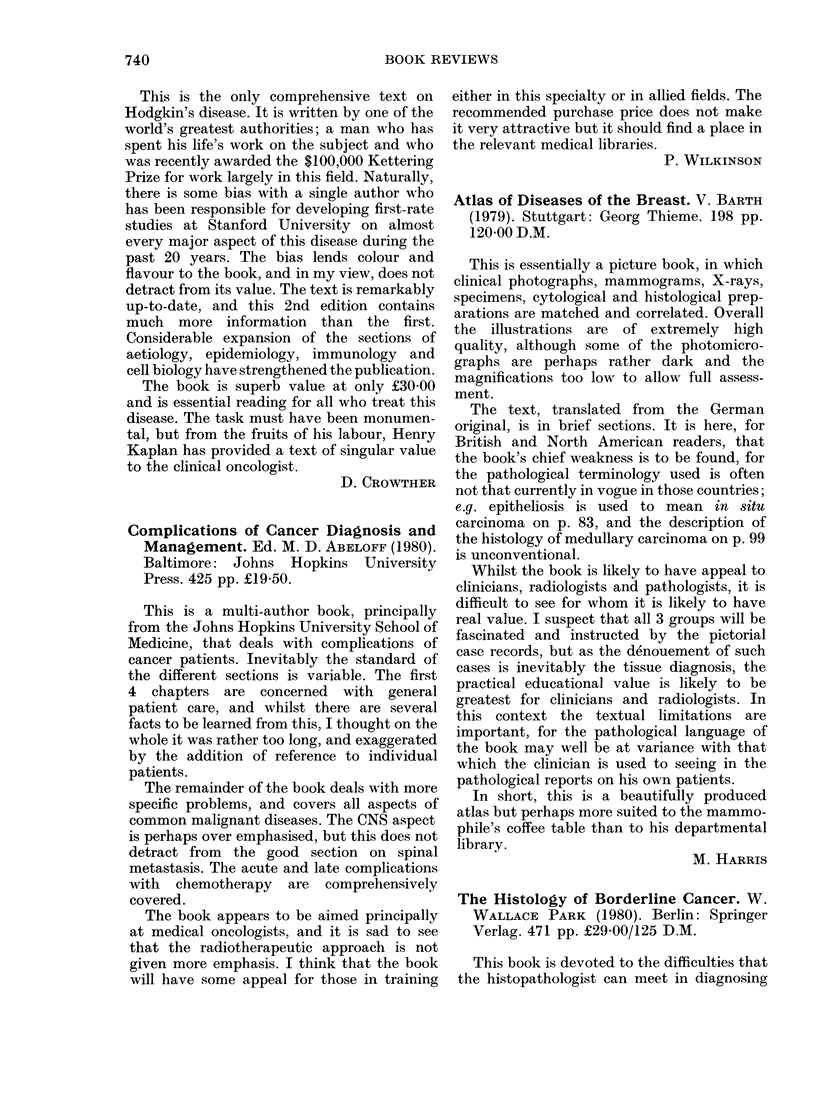# Complications of Cancer Diagnosis and Management

**Published:** 1981-05

**Authors:** P. Wilkinson


**Complications of Cancer Diagnosis and Management.** Ed. M. D. ABELOFF (1980). Baltimore: Johns Hopkins University Press. 425 pp. £19·50.

This is a multi-author book, principally from the Johns Hopkins University School of Medicine, that deals with complications of cancer patients. Inevitably the standard of the different sections is variable. The first 4 chapters are concerned with general patient care, and whilst there are several facts to be learned from this, I thought on the whole it was rather too long, and exaggerated by the addition of reference to individual patients.

The remainder of the book deals with more specific problems, and covers all aspects of common malignant diseases. The CNS aspect is perhaps over emphasised, but this does not detract from the good section on spinal metastasis. The acute and late complications with chemotherapy are comprehensively covered.

The book appears to be aimed principally at medical oncologists, and it is sad to see that the radiotherapeutic approach is not given more emphasis. I think that the book will have some appeal for those in training either in this specialty or in allied fields. The recommended purchase price does not make it very attractive but it should find a place in the relevant medical libraries.

P. WILKINSON